# Pediatric Case of Calcineurin Inhibitor-Induced Pain Syndrome Diagnosed During Cyclosporine Therapy for Aplastic Anemia

**DOI:** 10.7759/cureus.80602

**Published:** 2025-03-15

**Authors:** Tomohiro Tachikawa, Kuniaki Tanaka, Atsushi Iwai, Kiyohiro Kim, Ikuya Usami

**Affiliations:** 1 Pediatrics, Hyogo Prefectural Amagasaki General Medical Center, Hyogo, JPN; 2 Pediatric Hematology and Oncology, Hyogo Prefectural Amagasaki General Medical Center, Hyogo, JPN; 3 Pediatric Neurology, Hyogo Prefectural Amagasaki General Medical Center, Hyogo, JPN

**Keywords:** calcineurin inhibitor, cips, cyclosporine, immunosuppressive therapy, pain

## Abstract

A 15-year-old male with severe aplastic anemia was started on immunosuppressive therapy (IST) comprising antithymocyte globulin and cyclosporine (CsA), along with methylprednisolone and eltrombopag. Eight weeks after starting treatment, the CsA trough level increased to 269 ng/mL, coinciding with bilateral leg pain unresponsive to all analgesics, including fentanyl. MRI revealed diffuse high signal intensity areas on short tau inversion recovery (STIR) sequences in both thigh muscles. After discontinuing CsA and starting nifedipine, the pain improved, and an MRI taken 10 days post-discontinuation showed that the STIR high signal areas disappeared. Re-administration of CsA resulted in a recurrence of pain. Based on the clinical presentation and progression, the patient was diagnosed with calcineurin inhibitor-induced pain syndrome (CIPS). After switching from CsA to tacrolimus, the patient had no recurrence of pain. Although reports of CIPS in pediatric patients are extremely rare, it is important to consider CIPS as a differential diagnosis when a patient taking calcineurin inhibitors presents with severe pain in the lower limbs.

## Introduction

Calcineurin inhibitors (CIs), such as cyclosporine (CsA) and tacrolimus (Tac), are used widely as immunosuppressive therapy for organ transplantation or autoimmune diseases. Among their adverse effects, calcineurin inhibitor-induced pain syndrome (CIPS) is a clinical condition characterized by severe, intractable lower limb pain that occurs in association with the use of CIs [1]. This pain typically shows poor response to medications, including narcotic analgesics. CIPS can present with a variety of clinical features, including intractable lower limb pain, possible bone marrow or soft-tissue edema on MRI, and other nonspecific findings that may overlap with conditions such as infection, neuropathy, or musculoskeletal disorders. Estimates suggest that CIPS occurs in approximately 1.5-14% of post-transplant patients receiving CIs [2], although precise data in pediatric and non-transplant settings remain limited. In this report, we describe a pediatric case of CIPS that arose during CsA therapy for aplastic anemia, highlighting an atypical presentation and the need for clinicians to consider CIPS in the differential diagnosis when confronted with severe lower limb pain in patients undergoing calcineurin inhibitor therapy.

The data in the article were presented previously as a meeting abstract at the 65th Annual Meeting of the Japanese Society of Pediatric Hematology/Oncology (September 30, 2023).

## Case presentation

**Figure 1:**
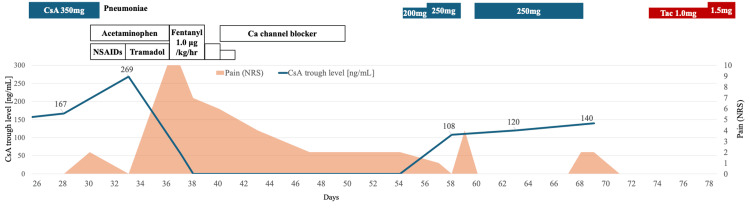
Clinical course after hospitalization for pneumonia and leg pain On Day 30 of immunosuppressive therapy (IST), the patient developed severe bilateral lower limb pain, coinciding with a rise in cyclosporine (CsA) trough levels (measured at 269 ng/mL on Day 33). CsA was discontinued on Day 33, and his pain gradually subsided. However, upon reintroducing CsA on Day 54, the patient experienced recurrent pain on Day 58. A second reintroduction on Day 61 led to another pain episode on Day 68. Ultimately, switching to tacrolimus on Day 71 resolved the pain, with no further recurrences.

A 15-year-old male presented with fatigue and was referred to our department after being diagnosed with pancytopenia. His white blood cell count was 1,600/µL (neutrophils 140/µL), hemoglobin was 7.6 g/dL, reticulocyte count was 10,000/µL, and the platelet count was 4,000/µL. Bone marrow biopsy showed a hypoplastic marrow without blasts but with small megakaryocytes. He was diagnosed with aplastic anemia. A matched related donor was not found, so he was started on immunosuppressive therapy (IST) with methylprednisolone, antithymocyte globulin, CsA, and eltrombopag. CsA dosing was adjusted to maintain trough levels between 150-250 ng/mL and C2 levels > 600 ng/mL. The patient developed leg pain on Day 30 of IST, and pneumonia on Day 32, leading to discontinuation of CsA and hospitalization. The clinical course after hospitalization is shown in Figure 1. He was treated with non-steroidal anti-inflammatory drugs (NSAIDs) for fever and pain. This was followed by an increase in creatinine levels from 0.79 mg/dL to 1.30 mg/dL. Despite discontinuation, the CsA concentration was still high, at 269 ng/mL, on Day 33. Blood test results on Day 33 are shown in Table 1.

**Table 1:** Blood test results on Day33 of immunosuppressive therapy (IST) PT: prothrombin time, INR: international normalized ratio, APTT: activated partial thromboplastin time, AST: aspartate aminotransferase, ALT: alanine aminotransferase, LD: lactate dehydrogenase, BUN: blood urea nitrogen

Test	Unit	Case	Reference
Leukocyte count	/µL	1300	3300–8600
Neutrophils	/µL	420	
Hemoglobin	g/dL	6.0	13.7–16.8
Reticulocyte	/µL	10000	
Platelet count	/µL	4000	158000–348000
PT-INR		1.08	
APTT	sec	39.2	23.0–36.0
D-dimer	μg/mL	3.0	<1
Total bilirubin	mg/dL	1.1	0.4–1.5
AST	U/L	23	13–30
ALT	U/L	19	10–42
LD	U/L	221	124–222
Creatine kinase	U/L	77	59–248
BUN	mg/dL	21.9	8–20
Creatinine	mg/dL	1.15	0.65–1.07
Glucose	mg/dL	99	73–109
C-reactive protein	mg/dL	0.11	0.00–0.14
Serum sodium	mEq/L	136	138–145
Serum potassium	mEq/L	4.4	3.6–4.8
Serum calcium	mg/dL	8.4	8.8–10.1
Serum iron	μg/dL	278	40–188
Ferritin	ng/mL	1954	13–277

**Figure 2:**
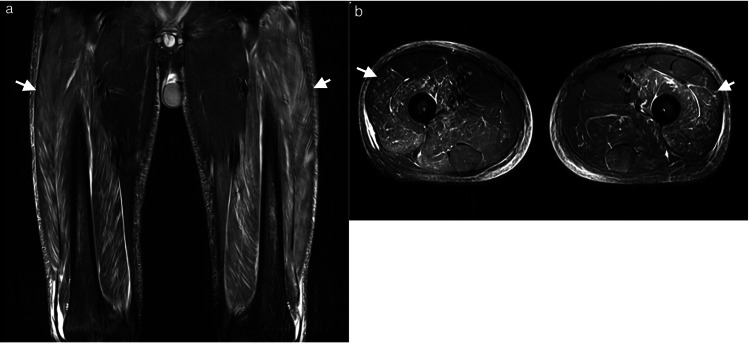
MRI findings on IST Day 40 (8 days after CsA discontinuation) (a) Short tau inversion recovery (STIR) coronal, (b) STIR axial Widespread STIR high signal areas in both thighs (white arrows), sparing the adductor muscles, with no bone abnormalities

Leg pain was characterized by spontaneous pain and tenderness, predominantly on the flexor side of both thighs and lower legs. Muscle strength testing showed decreased strength, notably in the distal muscles, and predominantly on the right side, where right foot drop was observed. The severe pain persisted despite administration of various analgesics, including acetaminophen, NSAIDs, tramadol, and fentanyl, leading to significant agitation. A head MRI, spine MRI, and cerebrospinal fluid test were normal. Given the clinical course and lack of response to analgesics, CIPS was suspected, and an MRI of the thighs was performed on Day 40 of IST (Figure 2). The MRI revealed widespread short tau inversion recovery (STIR) high signal areas in both thighs, sparing the adductor muscles, with no bone edema. A blood test showed a creatine kinase level of 685 U/L, which was a mild elevation not consistent with the symptoms, making myositis unlikely.

**Figure 3:**
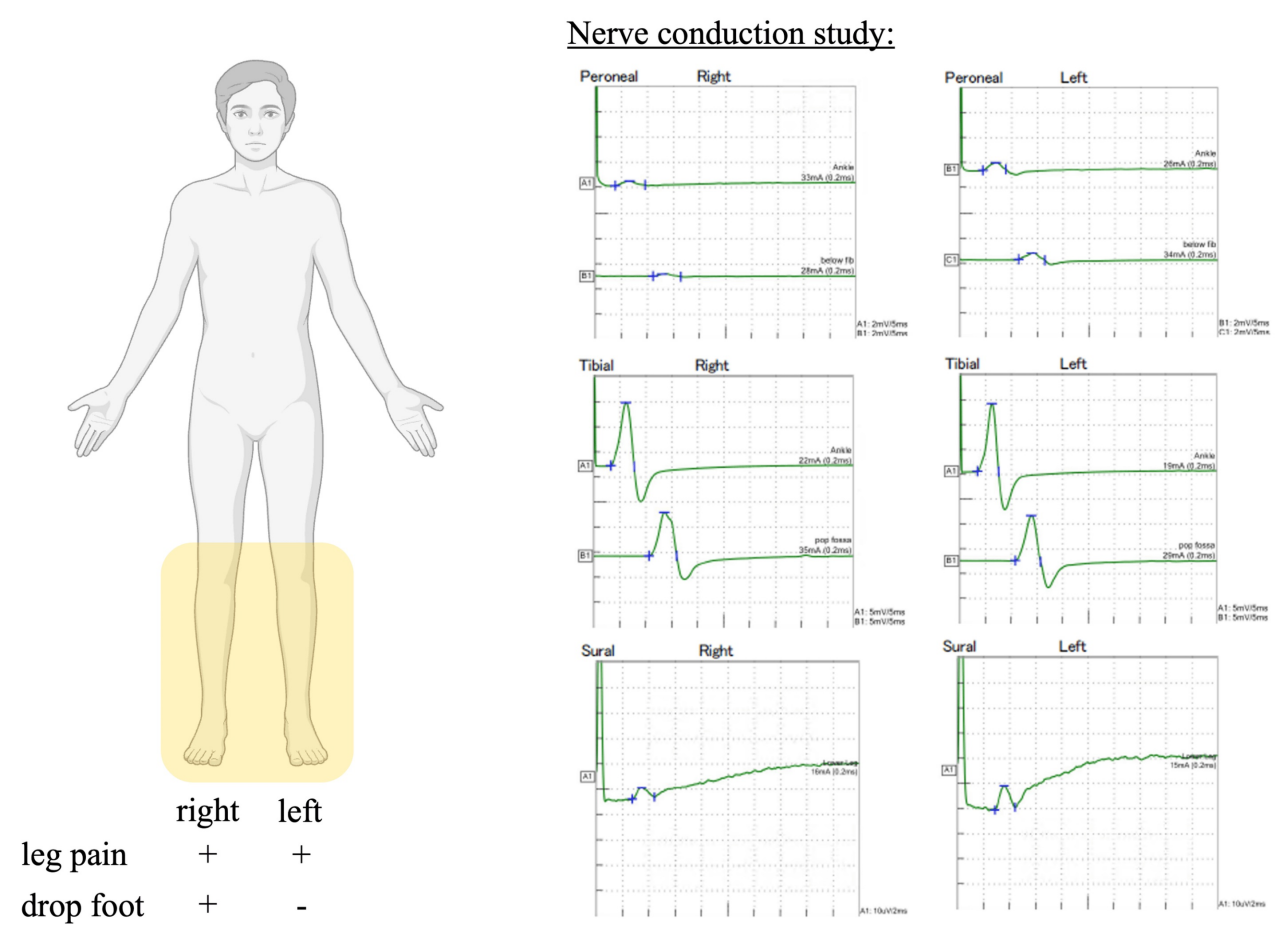
Nerve conduction studies (NCS) on Day 49 of IST (17 days after discontinuation of CsA) NCS data revealed reduced amplitudes in the right peroneal and right sural nerves, with no decrease in conduction velocity, consistent with the side where drop foot was observed. Image created in BioRender. Tanaka, K. (2025). https://BioRender.com/f50n286

**Figure 4:**
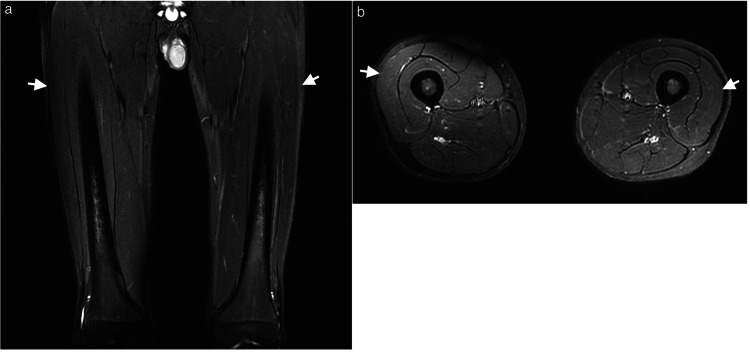
MRI findings on IST Day 50 (18 days after CsA discontinuation) (a) Short tau inversion recovery (STIR) coronal, (b) STIR axial The STIR high signal areas in both thighs had disappeared (white arrows). IST: immunosuppressive therapy; CsA: cyclosporine

Without reintroducing CsA, the initiation of oral calcium channel blockers led to a gradual improvement in the pain. Nerve conduction studies (NCS) on Day 49 of IST suggested axonal damage, particularly on the right side (Figure 3). An MRI on Day 50 revealed the disappearance of the STIR high signal areas in both thighs (Figure 4).

After the pain resolved, CsA was reintroduced on Day 54, but the pain returned on Day 58. After confirming that the pain had resolved again, CsA was reintroduced on Day 61; however, the pain returned on Day 68. Given this repeated correlation between CsA reintroduction and pain recurrence, with subsequent improvement upon cessation, alternative diagnoses such as avascular necrosis, myositis, and neuropathy became less likely, further supporting CIPS.

Based on our findings, several key diagnostic clues of CIPS emerged:

Medication Correlation

Symptom onset and recurrence were closely tied to CsA levels.

Pain Characteristics

Severe bilateral lower limb pain that was unresponsive to multiple analgesics (including opioids).

MRI Findings

STIR high-signal changes in soft tissue that resolved after discontinuing calcineurin inhibitors.

Laboratory Findings

Mild CK elevation without significant inflammatory markers or evidence of infection or autoimmune muscle disorder.

After switching to tacrolimus on Day 71, the patient had no further episodes of pain, but blood cell recovery was not achieved. Six months later, a cord blood transplant was performed, and engraftment was successful. Tacrolimus was used as the post-transplant immunosuppressant, without recurrence of CIPS. A chronological summary of these key diagnostic data and interventions is presented in Table 2.

**Table 2:** Chronological summary of key diagnostic data and interventions IST: immunosuppressive therapy, STIR: short tau inversion recovery, NCS: nerve conduction studies, CsA: cyclosporine, Tac: tacrolimus, CIPS: calcineurin inhibitor-induced pain syndrome

Day of IST	Major Clinical Events	Key Diagnostic Findings	Intervention / Outcome
30	Onset of severe bilateral lower limb pain	—	—
32	Pneumonia diagnosed	—	Hospitalization
33	Pain worsens	CsA trough: 269 ng/mL	CsA discontinued
40	Thigh MRI performed	MRI: Diffuse STIR high-signal areas in muscles (suggestive of CIPS)	CsA remains discontinued
49	NCS performed	NCS: Axonal damage (right side)	Findings support CIPS with neuropathic involvement
50	Repeat MRI	MRI: STIR changes resolved	Clinical improvement
54	Reintroduction of CsA	—	Pain recurrence on Day 58
61	Second CsA reintroduction	—	Pain recurrence on Day 68
71	Switch from CsA to Tac	—	Pain resolved

## Discussion

CIPS, first reported by Grotz et al. in 2001 [1], is a syndrome that causes severe pain in both lower limbs during CI use. It is reported primarily after solid organ transplantation, although it is rare, occurring in only 1.5-14% of post-transplant cases [2]. MRI findings, such as marrow edema or surrounding soft tissue swelling, can be indicative of CIPS [3]. Treatment primarily involves reducing or discontinuing the causative drug. Pain medications, including narcotic analgesics, are reportedly ineffective, but calcium channel blockers, pregabalin, and lidocaine show some success [1,4,5].

Pediatric presentations are rare, with the literature primarily documenting cases following organ or hematopoietic stem cell transplantation (Table 3). To the best of our knowledge, this case represents the second report of CIPS occurring in a pediatric patient that has not undergone transplantation. Given the absence of prior pediatric reports in non-transplant cases and the lack of bone marrow edema on MRI, this case was an atypical presentation. Consequently, we approached the diagnosis with significant caution. However, the re-emergence of pain after two separate re-initiations of CsA led us to a definitive diagnosis of CIPS. Considering that the initial MRI findings had disappeared completely from the second MRI image, the condition can resolve quickly. Since the first MRI was performed eight days after cessation of CsA administration, it is possible that the bone marrow edema improved prior to MRI acquisition.

**Table 3:** Previous reports of pediatric cases of calcineurin inhibitor-induced pain syndrome (CIPS) CIs: calcineurin inhibitors, Tac: tacrolimus, CsA: cyclosporine, PSL: prednisolone, CCB: calcium channel blocker, AML: acute myeloid leukemia

Authors	Reported Year	Age	Underlying Disease	CIs	MRI Findings	Treatment
Malat et al. [6]	2002	13	Wilson's disease → Liver transplant	Tac	-	Tac dose reduction
Nishikawa et al. [7]	2009	10	Aplastic anemia → Bone marrow transplant	Tac	Bone edema	Improvement with reduction in PSL
Lavoratore et al. [8]	2009	6	AML → Cord Blood Transplant	CsA	Bone marrow edema, High signal areas in soft tissue	Switch from CsA to Tac + CCB Discontinuation of Tac
Prates et al. [9]	2012	7	Renal failure → Kidney transplant	Tac	-	Switch from Tac to Sirolimus
Ayyala et al. [10]	2016	2	β thalassemia major →Bone marrow transplant	Tac	Bone marrow edema, High signal areas in soft tissue	Discontinuation of Tac
Nickavar et al. [11]	2014	10	Nephrotic syndrome	CsA	Bone marrow edema, High signal areas in soft tissue	Bone decompression (drilling)
This case	2023	15	Aplastic anemia	CsA	High signal areas in surrounding soft tissue	Switch from CsA to Tac

The exact mechanism that underlies CIPS is unclear; however, it is thought to be due to CI-mediated induction of inflammatory mediators and endothelial activation markers, causing vascular disturbance or activation of NMDA receptors, leading to hypersensitivity and pain [12,13]. It is suggested that there is a correlation between CsA serum concentration and the onset of CIPS [1]. The occurrence of CIPS in this non-transplant case may be attributable to several factors: administration of immunosuppressive therapy (as in other transplant cases) and use of NSAIDs during pneumonia, which may have caused renal impairment and resulted in an elevated serum concentration of CsA. Furthermore, it remains hypothetical that pneumonia could have triggered the production of inflammatory mediators and subsequent endothelial cell damage, a process requiring further investigation.

The NCS data suggested axonal damage and could also explain the right foot drop. This finding suggests a neuropathic component may be present in CIPS, potentially due to vascular disturbance. To the best of our knowledge, there are no previous reports of similar findings. Future cases should consider performing NCS to detect nerve involvement, as it may help establish diagnostic criteria for CIPS and guide the appropriate neurologic supportive care.

In this case, calcium channel blockers were clearly not effective, and cessation of CsA was necessary for pain relief. Although switching from CsA to tacrolimus is associated with symptom recurrence [14], this case was managed successfully with tacrolimus. Switching CI types may be a worthwhile strategy when CIPS is diagnosed. Although we did not administer pregabalin in this case, existing evidence indicates that CIs increase glutamate receptor activity in the spinal cord, and that inhibiting this activity at the spinal level reduces pain hypersensitivity in animal models [15]. Based on these findings, we believe that pregabalin could be a worthwhile therapeutic option.

It is anticipated that the use of CIs such as CsA and Tac will continue to increase for various indications, and so the incidence of CIPS is expected to rise. Further accumulation of case reports will help elucidate the pathophysiology, risk factors, and treatment options for this condition.

## Conclusions

Our case underscores the importance of considering CIPS as a differential diagnosis of severe lower limb pain in pediatric patients receiving CI, even when they are not transplant recipients. CIPS is an infrequent but impactful complication that can have a marked adverse effect on quality of life due to severe, treatment-resistant pain. Early recognition of CIPS in patients undergoing calcineurin inhibitor therapy is crucial, particularly because the use of these agents expands beyond transplant medicine to the treatment of autoimmune disorders and other diseases. In addition, our NCS findings suggesting axonal damage may be useful for the diagnosis of CIPS, particularly in atypical cases. Further case studies are essential to delineate the pathophysiology, identify risk factors, and optimize treatment protocols for CIPS. By increasing the repository of knowledge through case reporting and research, we can improve patient outcomes and inform clinical strategies for this debilitating syndrome.
